# An Atypical Presentation of Seronegative Rheumatoid Arthritis

**DOI:** 10.7759/cureus.36929

**Published:** 2023-03-30

**Authors:** Vineeta A Ramnauth, Patrick Rooney

**Affiliations:** 1 Neuroscience, Physiology and Behavioral Sciences, St. George's University, St. George, GRD; 2 Rheumatology, University Health Services, St. George's University, St. George, GRD

**Keywords:** seronegative, acr/eular classification, anti-citrullinate protein antibody, rheumatoid factor, rheumatoid arthritis

## Abstract

The American College of Rheumatology/European League Against Rheumatism (ACR/EULAR) 1987 classification for rheumatoid arthritis (RA) focuses on four main clinical findings without emphasizing biomarker serology. On the other hand, the updated ACR/EULAR 2010 classification relies more on acute-phase reactants and biomarker serology. While a positive rheumatoid factor (RF) and positive anti-citrullinated protein antibody (ACPA) are specific for RA, at least 15%-25% of patients are seronegative. Given that the ACR/EULAR 2010 classification is more likely to miss these seronegative patients, clinical judgment is important while assessing patients to avoid delays in diagnosis and onset of treatment.

## Introduction

Rheumatoid arthritis (RA) is an autoimmune disease with variable clinical presentations. When untreated or if there is a delay in the onset of treatment, multiple joints, and other organ systems may develop progressive inflammatory destruction. Global prevalence for RA ranges from 0.24% to 1%, with a higher prevalence in urban regions (0.69%) when compared to resource-limited rural areas (0.48%) [1,2]. This variability suggests inconsistencies in access to health care and the impact of different environments on RA incidence and detection [2]. In Latin American and the Caribbean (LAC) region, the prevalence of RA ranges from 0.4% to 1.6%, with a five-times more likelihood of the disease among women and an earlier onset of symptoms and complications [3,4].

Typical features of RA

The "classic" patient profile for RA is a woman between 50-75 years with a history of tobacco use who presents with pain and swelling or stiffness in at least seven symmetrical joints of the hands or feet [5,6]. The joint pain worsens in the morning, lasting more than one hour and improving with movement [5,7]. Associated constitutional symptoms include fatigue, malaise, and weight loss [5]. During the earlier stages of the disease, physical examination shows tender, soft tissue swelling around symmetrical joints of the hands or feet. Advanced or severe disease presents with trigger fingers, ulnar deviation, swan-neck deformities, boutonnière deformities, and firm rheumatoid nodules [8,9]. The most common extra-articular manifestation of RA is cardiovascular disease [9,10].

Making the diagnosis

Diagnosing RA requires algorithms based on clinical features and laboratory studies [6]. Elevated acute-phase reactants such as C-reactive protein (CRP) and erythrocyte sedimentation rate (ESR) and positive biomarker serology for rheumatoid factor (RF) and anti-citrullinated protein antibody (ACPA) usually indicate RA. A 10-year prospective study in France found that 26% of patients with RA had elevated ESR, 39% had positive CRP, 39% had positive ACPA, and 45% had elevated RF [5]. In comparison, a meta-analysis showed higher rates of positive RF (69%) and positive ACPA (67%) [11]. Between 15%-25% of patients with clinical symptoms of RA have negative RF and negative ACPA, with 30%-45% having negative RF during the early stages of their illness [11,12].

Radiological findings vary depending on the stage of the disease. During the earlier phases, joint effusions and soft tissue swellings with osteopenia and normal joint spaces can be detected [9,11]. With worsening of the disease, radiological findings show damage to joint cartilage, narrowing of the joint space, erosion of the capsular margins, subluxation, and ankylosis [9,11]. The American College of Rheumatology/European League Against Rheumatism (ACR/EULAR) has developed criteria for diagnosing RA using clinical and laboratory findings [13]. 

We report an atypical case of RA in a man who does not meet the ACR/EULAR 2010 classification, yet has a clinical history and late-onset joint changes suggestive of RA. Our objective for reporting this case is to demonstrate the importance of a physician's clinical judgment while using guidelines for diagnosing and managing patients with atypical presentations.

## Case presentation

A 60-year-old man presented to the rheumatologist with undiagnosed joint pain and stiffness. It began approximately twenty years ago with stiffness in the toes that progressed to pain in the neck, knees, wrists, sternum, spine, and ankles. The pain is a burning sensation that radiates in waves away from the joint and worsens at rest and initial movement. He occasionally experiences flares of pain in the knees, limiting mobility. During these flares, the pain is unresponsive to analgesics such as non-steroidal anti-inflammatory drugs (NSAIDs). Over the past five years, the patient noticed changes in his fingers and described them as looking twisted. There was no fever, malaise, or unintentional weight loss; however, he reported evening fatigue. The patient was previously investigated for the cause of his joint pain and received no diagnosis of osteoarthritis, gouty arthritis, or RA. He used multivitamins, low-dose aspirin, folic acid, Vitamin B complex, and omega-3 daily. His eldest brother had bilateral knee replacement surgery for degenerative osteoarthritis, and two older siblings have undiagnosed joint pains. He had a strong family history of atopy but no known history of autoimmune disorders. He did not consume alcohol, use illicit drugs or tobacco products, and has no history of trauma. He consumed a pescatarian diet.

Physical examination showed an afebrile, hydrated, and hemodynamically stable man in no apparent painful distress. The fourth digit of his right hand had a swan neck deformity, while the left 4th and 5th digits had both swan neck deformity and ulnar deviation. There was no boutonniere deformity, Heberden's nodes, or rheumatoid nodules. No muscular atrophy, joint swellings, joint tenderness, or effusions were present. On auscultation, there were clear breath sounds and no extracardiac sounds. There was no abdominal tenderness/distension or abnormalities in the central nervous system.

Investigations

Previous laboratory studies were done to investigate the cause of his joint pains, and all results were within the normal reference range. The most recent laboratory studies requested by his family physician within the past year are shown in Table 1.

**Table 1:** Summary of recent laboratory studies.

Test	Result	Laboratory reference range
Cyclic citrullinated peptide Ig G	2.8 U/mL	negative <20
Rheumatoid factor	5.2 U/mL	negative <25
Erythrocyte sedimentation rate	9 mm/hr	0 – 15
Alkaline phosphatase	66 U/L	30 - 120
Alanine transaminase	18 U/L	0 - 40
Aspartate aminotransferase	17 U/L	0 - 37
Calcium	9.2 mg/dL	8.10 – 10.4
Uric acid	3.3 mg/dL	3.4 – 7.0
Antinuclear antibody	0.68 units	0 – 1.0

**Figure 1:**
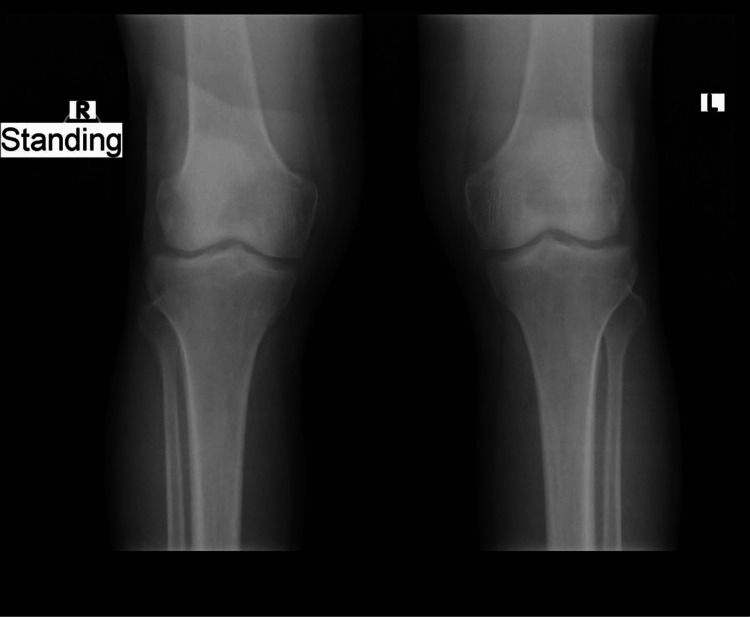
Knee radiograph showing normal articulation of bones forming the knee joint.

**Figure 2:**
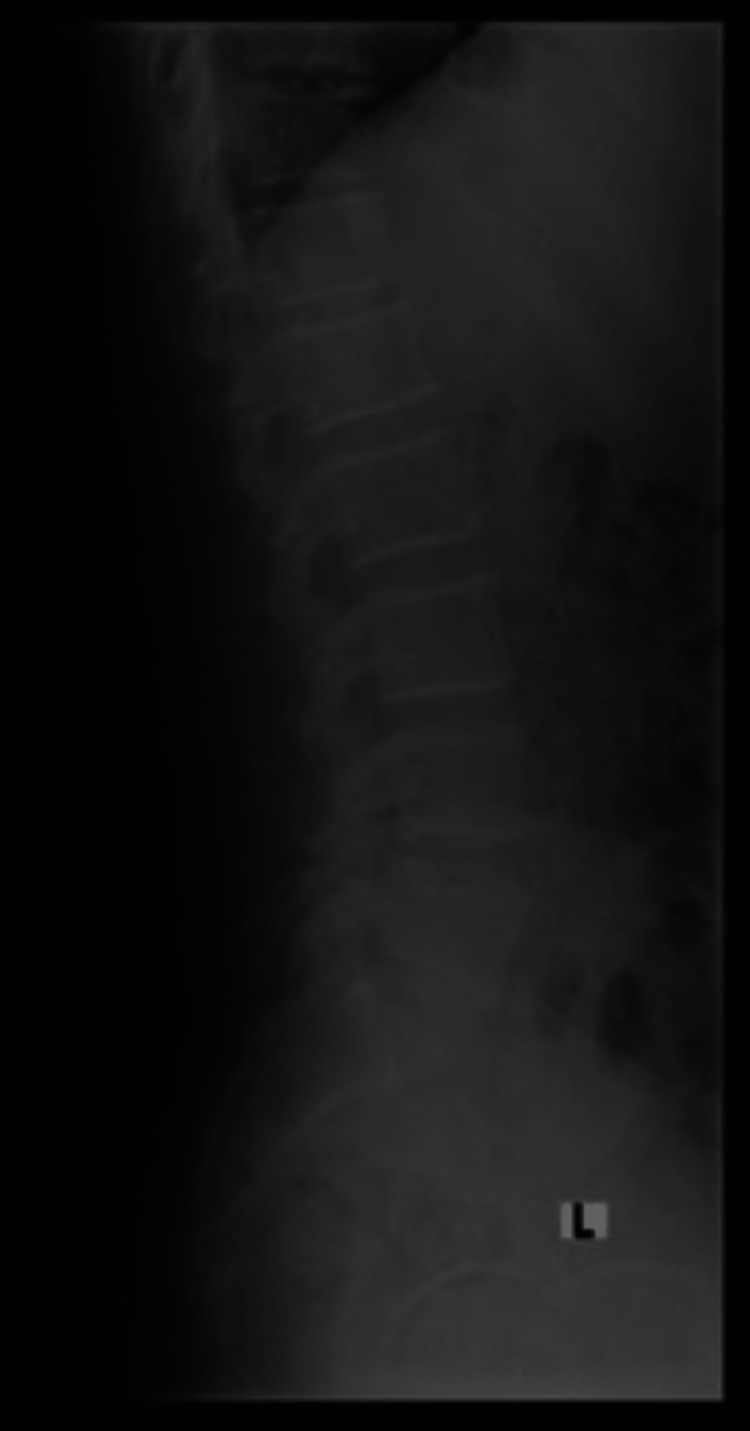
Thoracic radiograph showing loss of spinal curvature.

Images of the knees and thoracolumbar region were taken within the past year and are shown in Figure 1, and Figure 2, respectively.

**Figure 3:**
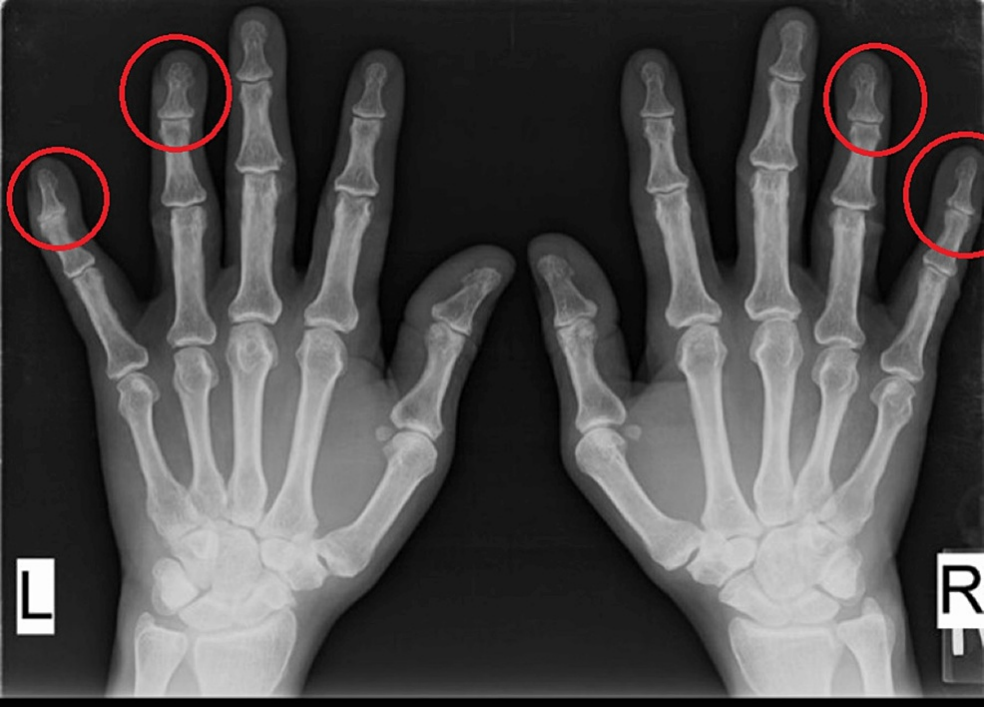
Radiograph of the hands showing flexion of the terminal phalanx of the 5th finger of the left hand and normal bones.

**Figure 4:**
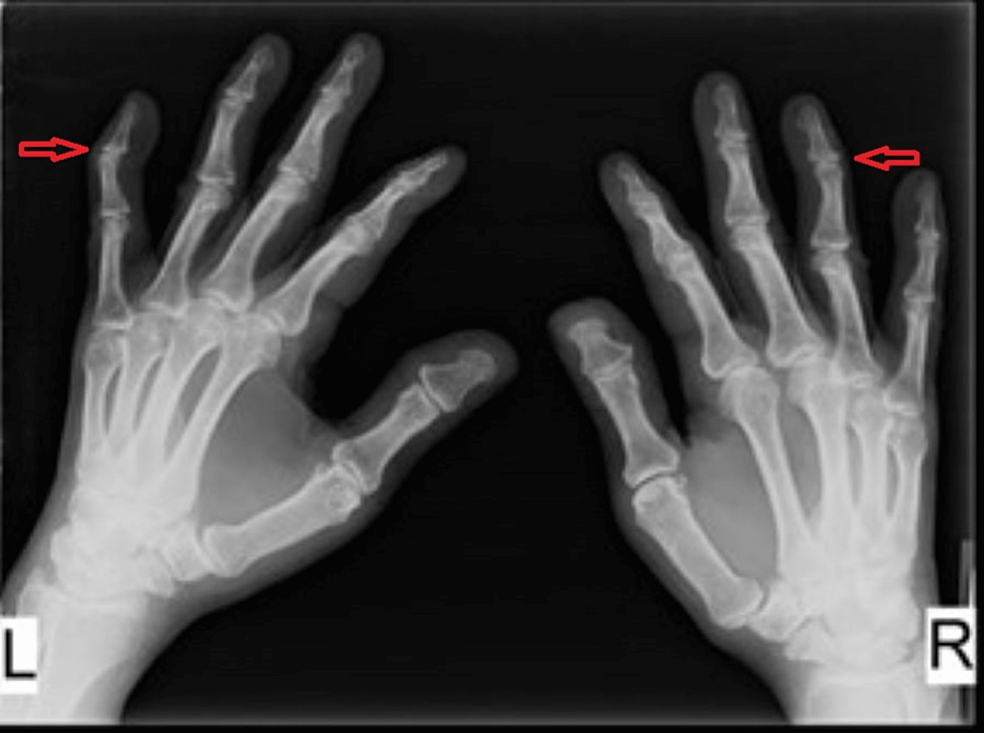
Radiograph of the hands showing flexion of the terminal phalanx of the 5th finger of the left hand with mild thickening of extensor soft tissue at the level of distal phalanx of the left 5th finger.

Radiographs of the hands showed flexion of the terminal phalanx and soft tissue thickening shown in Figure 3 and Figure 4, respectively.

Repeated acute-phase reactants and biomarker serology were in the normal reference range (Table 2).

**Table 2:** Repeated biomarker serology.

Test	Result	Laboratory reference range
Erythrocyte sedimentation rate (ESR)	5 mm/hr	0-15
Antinuclear antibody (ANA)	0.68 mm/dL	0-1
Rheumatoid factor (RF)	<20 IU/mL	<20

Differential diagnosis

The differential diagnoses for chronic afebrile polyarticular joint pain include noninflammatory (osteoarthritis, calcium pyrophosphate dihydrate crystal deposition disease) and rheumatologic diseases (RA, systemic lupus erythematosus, spondyloarthropathy, psoriatic arthritis). Infectious causes (gonococcal arthritis, Lyme disease, bacterial endocarditis, rubella, hepatitis, HIV, parvovirus) and postinfectious causes (enteric, urogenital, rheumatic fever) are unlikely without a history of febrile illness. A comparison of the differential diagnosis for chronic afebrile polyarticular joint pain and the patient's findings is shown in Table 3.

**Table 3:** Differential diagnosis for chronic afebrile polyarticular joint pain.

Differential Diagnosis	Comment	Patient's Findings
Osteoarthritis	Spares the wrist and MCP joints Stiffness <30 minutes Pain relieved with rest Crepitus + osteophytes Narrowed joint space	Wrist and MCP joints involved Pain worse at rest No crepitus or osteophytes Joint space maintained
Calcium pyrophosphate dihydrate crystal deposition disease	Pain, swelling, tenderness, erythema Involves wrist and MCP joints. Calcium crystals in radiograph	No swelling or erythema No crystals on radiograph
Spondyloarthropathy	Involves lower limbs, spine, sacroiliac joints Starts before 40 years as back pain then progresses	Pain began in feet and hands in late 40s
Polymyalgia rheumatica	Proximal muscle pain and stiffness around shoulder and hip Spares small joints	Small joints involved
Systemic lupus erythematosus	Malar rash, photosensitivity, discoid skin lesions, alopecia Double-stranded DNA	None present

Outcome and follow-up

This patient had a history of afebrile polyarticular joint pain and stiffness relieved with activity, late-onset joint changes in the hands, and negative acute-phase reactants and biomarker serology. According to the ACR/EULAR 2010 classification, he did not meet the criteria for RA, but the case was clinically suggestive of seronegative RA. The initial three-month trial of methotrexate 10mg once weekly was started; the patient was followed up to assess subjective pain control, progression of joint changes, and any changes in laboratory studies. Since starting the methotrexate, the patient reported fewer pain episodes and minimal pain flairs. He exercises for at least 45 minutes daily and now eats a strictly vegan diet. There have been no further joint changes, and his laboratory studies show mild bone marrow suppression.

## Discussion

RA is diagnosed with the aid of the ACR/EULAR 2010 classification. This classification has a maximum of five points allocated to joint distribution, four points to acute-phase reactants/serology, and one point based on the duration of symptoms. Using these criteria, a patient requires six or more points to be diagnosed with RA (Table 4).

**Table 4:** American College of Rheumatology/European League Against Rheumatism (ACR/EULAR) 2010 rheumatoid arthritis classification. Joint distribution refers to findings during physical examination showing swollen or tender joints [13]. Small joints refer to metacarpophalangeal (MCP) joints, proximal interphalangeal (IP) joints, second to fifth metatarsophalangeal (MTP) joints (excluding the first), wrists and thumb interphalangeal joints [13]. Large joints refer to shoulders, elbows, hips, knees, and ankles [13]. ACPA = anti-citrullinated protein antibody; CRP = C-reactive protein; ESR = erythrocyte sedimentation rate; RF = rheumatoid factor.

Attribute	Attribute Characteristic	Score
Joint distribution	1 Large joint	0
	2–10 Large joints	1
	1–3 Small joints (large joints not counted)	2
	4–10 Small joints (large joints not counted)	3
	>10 Joints (at least one small joint)	5
Serology	Negative RF and negative ACPA	0
	Low positive RF or low positive ACPA	2
	High positive RF or high positive ACPA	3
Symptom duration	<6 weeks	0
	>6 weeks	1
Acute phase reactants	Normal CRP and normal ESR	0
	Abnormal CRP or abnormal ESR	1

In comparison, the earlier ACR/EULAR 1987 classification gave more importance to the clinical features of RA, such as joint involvement, symmetry, and the presence of rheumatoid nodules, with less emphasis on biomarkers [14] (Table 5). It also used only one biomarker, RF, instead of the four acute-phase reactants/serology markers (RF, ACPA, ESR, and CRP) used by the ACR/EULAR 2010 classification [15].

**Table 5:** The American College of Rheumatology/European League Against Rheumatism (ACR/EULAR) 1987 classification for rheumatoid arthritis.

Criteria	Definition
Morning stiffness	Morning stiffness in and around the joints, lasting at least 1 hour before maximal improvement.
Arthritis in three or more joint areas	At least three joint areas simultaneously have had soft tissue swelling or fluid (not bony overgrowth alone) observed by a physician. The fourteen possible areas are right or left PIP, MCP, wrist, elbow, knee, ankle, and MTP joints.
Arthritis of hand joints	At least one area is swollen (as defined above) in a wrist, MCP, or PIP joint.
Symmetric arthritis	Simultaneous involvement of the same joint areas (as defined in 2) on both sides of the body (bilateral involvement of PIPs, MCPs, or MTPs is acceptable without absolute symmetry).
Rheumatoid nodules	Subcutaneous nodules over bony prominences, extensor surfaces, or in juxta-articular regions are observed by a physician.
Serum rheumatoid factor	Demonstration of abnormal amounts of rheumatoid serum factor by any method for which the result has been positive in <5% of normal control subjects.
Radiological changes	Radiographic changes typical of rheumatoid arthritis on posteroanterior hand and wrist radiographs must include erosions or unequivocal bony decalcification localized in or most marked adjacent to the involved joints (osteoarthritis changes alone do not qualify).

The ACR/EULAR 1987 classification aimed to distinguish RA from other causes of joint pain, while the updated ACR/EULAR 2010 classification was made to precisely diagnose RA [15]. There are, however, some limitations to the ACR/EULAR 2010 classification. Seronegative patients, otherwise detected by the ACR/EULAR 1987 classification, are missed by the ACR/EULAR 2010 classification [16,17]. Among seronegative patients with RA, women were more likely to have delays in diagnosis using the ACR/EULAR 2010 classification [18].

This clinical case presents an atypical scenario of a middle-aged man with prior joint pain and stiffness with no swelling or tenderness during examination by the rheumatologist. All acute-phase reactants and biomarker serology were negative, and the patient did not meet the criteria of either the ACR/EULAR 1987 or 2010 classification to be diagnosed with RA. Clinical findings did not suggest osteoarthritis or other causes of afebrile polyarticular joint pain. As the disease progressed over time, joint deformities in the hands characteristic of RA began to appear and a presumptive diagnosis of seronegative RA was considered. After a three-month trial of methotrexate, a decrease in pain flares without further progression in joint changes made the diagnosis of RA plausible.

When using the ACR/EULAR 2010 classification, without positive biomarker serology or acute-phase reactants, physicians are cautious about diagnosing RA despite the presence of symmetrical joint pain. Other causes of afebrile polyarthritis are explored, with possible delays in the diagnosis and onset of treatment. In resource-limited rural regions, biomarkers might be expensive or unavailable, and physicians may rely on clinical features to make their diagnoses. In cases of atypical presentations of RA or late onset of symptoms, delays in diagnosis and treatment onset can occur when considering only the classic clinical features of RA.

RA is generally difficult to diagnose because of the numerous overlapping symptoms. Biomarker serology was meant to assist with reliably diagnosing RA; however, in seronegative patients, other causes of arthritis will be ruled out before a diagnosis is made. Seronegative RA reflects the absence of commonly tested biomarkers, however, other biomarkers may be elevated. If the patient can afford other types of biomarkers and they are available, anti-RA 33, antikeratin antibodies, and antiperinuclear factors can be used in otherwise seronegative cases [19]. Anti-RA 33 is present in approximately 33% of persons with RA and can be useful in seronegative patients [20].

While the ACR/EULAR 2010 classification does not specifically include the use of MRI or ultrasound in its criteria, these imaging modalities can be used when there is diagnostic uncertainty [21,22]. In the absence of biomarkers or delayed clinical findings, imaging studies such as high-frequency ultrasound and magnetic resonance imaging (MRI) are useful for the early detection of RA. Some studies consider the use of MRI or ultrasound findings superior to clinical examinations when diagnosing early RA [22]. Radiographs typically detect bone changes that are evident during the later stages of the disease. An ideal imaging modality is the use of MRI scans that are less user-dependent and able to detect early bone changes such as erosions, and inflammation (synovitis, tenosynovitis, and bursitis) not otherwise shown on radiographs [23]. These early bone erosions are more likely to be detected on MRI scans within six months of the onset of RA in approximately 72% of persons when compared to 8%-40% using radiographs [23]. Hence, during the early stages of RA when clinical and serological findings are not yet apparent, MRI scans can detect signs of inflammation and bone changes. While an ideal and highly specific imaging modality, MRI scans are costly, not readily available in all regions, require a contrast medium to improve quality, and can only examine selected joints at a given time [21]. Therefore, in resource-limited regions where MRI scans might not be readily available or affordable, high-frequency ultrasound can be used as an acceptable alternative [23]. Using an ultrasound to detect early findings of RA is however limited by the quality of the machine used and the experience of the operator [23].

Physicians are likely to underdiagnose atypical RA if reliant on only clinical findings and the presence of biomarkers and acute-phase reactants. While the ACR/EULAR 2010 classification does not specify the use of imaging modalities such as MRI and ultrasound within its criteria, these have been shown to aid in making an early diagnosis of RA, especially when clinical and serological findings are inconclusive. There is merit though in recognizing the importance of physicians trusting their gut instinct about patients. Despite not being certain of the diagnosis based on the ACR/EULAR 2010 classification, there was a benefit to considering a trial period with medication in this case. 

## Conclusions

The ACR/EULAR 2010 classification is more likely to miss seronegative patients leading to delays in diagnosis and treatment onset. In this atypical presentation of RA without the classic history of joint pain and swelling or positive acute-phase reactants and biomarker serology, clinical judgment should not be ignored. Guidelines and algorithms should be used as aids rather than an all-or-none experience for physicians and patients. Although the ACR/EULAR 2010 classification does not specify the use of imaging modalities, MRI and high-frequency ultrasound can detect early signs of bone changes and inflammation during the early stages of RA, especially when clinical and serological findings are inconclusive.

Late-onset rheumatoid arthritis or patients with atypical clinical features likely receive late diagnosis and onset of treatment. Alternative biomarkers can be considered in patients who are seronegative to the typical biomarkers such as rheumatoid factor and anti-citrullinated protein antibody. Imaging modalities such as MRI and high-frequency ultrasounds should be considered when clinical and serological findings are inconclusive. MRI scans can detect early changes within six months of the onset of RA. Physicians should trust their clinical judgment, using guidelines and algorithms as aids to making their diagnosis.
